# A Virulent *Babesia bovis* Strain Failed to Infect White-Tailed Deer (*Odocoileus virginianus*)

**DOI:** 10.1371/journal.pone.0131018

**Published:** 2015-06-17

**Authors:** Massaro W. Ueti, Pia U. Olafson, Jeanne M. Freeman, Wendell C. Johnson, Glen A. Scoles

**Affiliations:** 1 USDA, ARS, Animal Disease Research Unit, Washington State University, Pullman, Washington, United States of America; 2 USDA, ARS, Knipling-Bushland United States Livestock Insects Research Laboratory, Kerrville, Texas, United States of America; Universidade Federal do Rio de Janeiro, BRAZIL

## Abstract

Wildlife are an important component in the vector-host-pathogen triangle of livestock diseases, as they maintain biological vectors that transmit pathogens and can serve as reservoirs for such infectious pathogens. *Babesia bovis* is a tick-borne pathogen, vectored by cattle fever ticks, *Rhipicephalus* spp., that can cause up to 90% mortality in naive adult cattle. While cattle are the primary host for cattle fever ticks, wild and exotic ungulates, including white-tailed deer (WTD), are known to be viable alternative hosts. The presence of cattle fever tick populations resistant to acaricides raises concerns regarding the possibility of these alternative hosts introducing tick-borne babesial parasites into areas free of infection. Understanding the *B*. *bovis* reservoir competence of these alternative hosts is critical to mitigating the risk of introduction. In this study, we tested the hypothesis that WTD are susceptible to infection with a *B*. *bovis* strain lethal to cattle. Two groups of deer were inoculated intravenously with either *B*. *bovis* blood stabilate or a larval extract supernatant containing sporozoites from infected *R*. *microplus* larvae. The collective data demonstrated that WTD are neither a transient host nor reservoir of *B*. *bovis*. This conclusion is supported by the failure of *B*. *bovis* to establish an infection in deer regardless of inoculum. Although specific antibody was detected for a short period in the WTD, the PCR results were consistently negative at multiple time points throughout the experiment and blood from WTD that had been exposed to parasite, transferred into naïve recipient susceptible calves, failed to establish infection. In contrast, naïve steers inoculated intravenously with either *B*. *bovis* blood stabilate or the larval extract supernatant containing sporozoites rapidly succumbed to disease. These findings provide evidence that WTD are not an epidemiological component in the maintenance of *B*. *bovis* infectivity to livestock.

## Introduction

Stable enzootic foci of arthropod-borne pathogens are successfully maintained as a result of the interaction between mammalian reservoirs and competent arthropod vectors [1,2]. The ability of the pathogen to infect both vertebrate and invertebrate hosts is essential for the spread of disease [3–5]. This complex cycle is exemplified by tick-borne pathogens such as *Babesia bovis* and *B*. *bigemina*, the causative agents of bovine babesiosis [6,7]. In endemically stable areas, female cattle fever ticks, *Rhipicephalus microplus* and *R*. *annulatus*, serve as competent vectors that acquire babesial parasites from the mammalian reservoir [7,8]. The parasites infect tick midgut epithelial cells where they complete transformation into kinetes. This is a critical step in the transmission cycle, as kinetes are transmitted transovarially and the resulting tick offspring are then capable of transmitting the pathogen to naïve hosts, maintaining the endemic cycle [3,4,6]. It is increasingly clear that wildlife, especially white-tailed deer (*Odocoileus virginianus*; WTD), play an important epidemiological role in sustaining cattle fever tick populations in the presence or absence of livestock [9–12]. Deer serve as viable alternative hosts for cattle fever ticks, and free-ranging WTD are capable of disseminating these ticks into areas previously free of the biological vector [12,13]. This is especially important in the southern region of the United States where WTD populations have increased and are gradually expanding in distribution. Further, the frequent movement of WTD between the U.S. and Mexico increases the likelihood of transporting cattle fever ticks into the U.S. from Mexico where bovine babesiosis is endemic [8]. Should these cattle fever ticks be infected with *B*. *bovis*, they could transmit to naïve cattle resulting in significant economic losses for the livestock industry. The role of WTD as reservoirs for *B*. *bovis* and *B*. *bigemina* is unclear.

Kuttler et al (1972) reported that WTD do not develop a detectable level of *Babesia* parasites upon either infestation with *R*. *annulatus* larval progeny originating from female cohorts reared on a *Babesia*-infected calf from Mexico or inoculation with blood from an infected bovine [14]. However, the *Babesia* spp. was unknown, parasite load being delivered to the deer was not quantified, and sensitive detection methods were not available for monitoring the experimental animals. More recently, molecular and serological analyses of samples from free-ranging WTD in Texas and northern Mexico implicated cervids as potential reservoirs of bovine babesiosis parasites [15–18]. The results suggested either transient babesial infection or cross reactivity to parasites that are capable of establishing an infection in deer. Due to the nature of the sample acquisition, the animals could not be monitored over time to distinguish between these possibilities.

In the current study, we addressed these issues and determined that a strain of *B*. *bovis* lethal to cattle failed to infect WTD via intravenous inoculation. Blood infected with *B*. *bovis* generated in a splenectomized calf or a larval extract supernatant containing *B*. *bovis* sporozoites from infected *Rhipicephalus* larvae did not infect WTD. In contrast, naïve steers inoculated intravenously with either *B*. *bovis* blood stabilate or the larval extract containing sporozoites rapidly succumbed to disease. We discuss our findings in the context of epidemiology, transmission and reservoir capacity for bovine babesiosis parasites.

## Materials and Methods

### Pathogen, ticks and animals

In this study, we used a highly transmissible strain of *B*. *bovis* that is lethal to cattle and for which there exists a complete and annotated genome [19]. This strain of *B*. *bovis* originated from a quarantined animal in Texas. The La Minita strain of *R*. *microplus* isolated from cattle on pasture in Starr County, TX was used to produce a larval extract supernatant containing *B*. *bovis* sporozoites. Previous studies demonstrated that this *R*. *microplus* colony is efficient in acquiring and transmitting *B*. *bovis* parasites to naïve cattle [3,4].

To determine the susceptibility of WTD to *B*. *bovis*, eight WTD doe fawns were hand raised at the Knipling-Bushland U.S. Livestock Insects Research Laboratory (KBUSLIRL) in tick-free facilities. Steers were used to verify the viability and infectivity of *B*. *bovis* parasitized erythrocytes or the larval extract containing sporozoites. Prior to use, all animals tested negative for *B*. *bovis* by immunoblots and PCR amplification targeting the 18S rRNA gene [15,20].

### Inoculum preparation

*Babesia bovis* parasitized erythrocytes were generated in a splenectomized calf [21] and used as the source of infection for the deer experiments. To prepare *B*. *bovis* blood stabilate, a splenectomized calf was infected intravenously with a lethal strain of *B*. *bovis*. When the parasitemia reached 1.1%, blood was collected, washed to remove white blood cells and cryopreserved using dimethyl sulphoxide in liquid nitrogen [21]. A larval extract supernatant containing *B*. *bovis* sporozoites was produced from infected tick larvae. Briefly, *R*. *microplus* larvae were applied under a cloth patch on a splenectomized naïve calf. When ticks molted to adults, approximately 10^7^ *B*. *bovis* parasitized erythrocytes were inoculated intravenously into the calf. Female ticks that fed to engorgement during an ascending parasitemia were collected to rear infected larvae. Egg masses from infected engorged female ticks were pooled and one gram of tick eggs per vial was incubated at 26°C with 96% relative humidity. After hatching, larvae were incubated at 15°C with 96% relative humidity [3,4].

To stimulate the development of *B*. *bovis* sporozoites in tick salivary glands [3,4], infected larvae from one gram of eggs were placed under a cloth patch on a naïve calf Ticks were forcibly removed after four days of larval feeding. The stimulation fed, infected larvae were incubated on ice for 30 min. Approximately 300 larvae were added to cold sterile phosphate buffered saline (PBS) and ground using a tissue homogenizer. Supernatants were pooled and centrifuged at 400g, 4°C for 10 min to remove tick debris. The pellets were discarded and the larval supernatant containing *B*. *bovis* sporozoites was used to challenge WTD.

### Challenge

Two groups of WTD were used to determine their susceptibility to *B*. *bovis*. The parasite levels in the inocula were determined by quantitative real time PCR [4,22]. Group 1 contained four 2-year old WTD and one intact steer as a control. Group 2 contained four 3-year old WTD and one intact steer as a control. Group 1 received *B*. *bovis* parasitized erythrocytes and group 2 received a larval extract containing *B*. *bovis* sporozoites produced from infected *R*. *microplus* larvae. The inocula were suspended in 10% homologous deer or bovine serum in Puck’s saline G and inoculated intravenously into the jugular vein.

### Infection status of animals

The infection status of WTD and control cattle was determined by monitoring for clinical signs of babesiosis including alteration in body temperature and packed cell volume (PCV). The infection status was confirmed by performing molecular and serological assays at multiple time points after parasite inoculation. Deer blood samples and sera were collected daily for the first two weeks of the study and weekly up to 84 days post-challenge. Genomic DNA was isolated from whole blood with the DNeasy Blood and Tissue Kit (Qiagen) and was used as template in a nested PCR targeting 18S rRNA, a multi copy gene [15,19]. It was predicted that the 18S rRNA outer primers (Nbab-1 forward, 5′-AAG CCA TGC ATG TCT AAG TAT AAG CTT TT-3′ and Nbab-1 reverse, 5′-CTT CTC CTT CCT TTA AGT GAT AAG GTT CAC-3′) would amplify a fragment of 1,600 bp [23]. PCR was carried out under the following conditions: 95°C for 5 min; 35 cycles of 95°C for 30 sec, 50°C for 30 sec, and 72°C for 1 min; final extension at 72°C for 5 min. The reaction was conducted in 32 μl containing 2 μl of extracted genomic DNA, 2.0 mM MgCl_2_, 200 μM dATP, dCTP, dGTP, dTTP; 1.0 μM of each primer set, and 1.3 U of FastStart Taq (Roche). It was predicted that the 18S rRNA inner primers (forward, 5’- AATCCTGACACAGGGAGGTAGTGAC-3’ and reverse, 5’-CTAAGAATTTCACCTCTGACAGT-3’) would amplify a fragment of 390 bp. Nested PCR was carried out under the following conditions: 95°C for 5 min; 35 cycles of 95°C for 30 sec, 65°C for 30 sec, and 72°C for 30 sec; final extension at 72°C for 5 min. The reaction was conducted in 30 μl containing 0.1 μl from the first reaction, 2.0 mM MgCl2, 200 μM dATP, dCTP, dGTP, dTTP; 1.0 μM of each primer set, and 1.25 U of FastStart Taq (Roche). DNA isolated from all collection dates were screened by PCR and ten replicate amplifications were conducted per animal per sampling date to capture low parasitemia levels. To determine the sensitivity of the nested PCR, serial dilutions of *B*. *bovis* infected erythrocyte cultures were performed. Briefly, cultures with 10^7^ infected erythrocytes per ml of blood were diluted serially 10 fold in uninfected bovine blood to a final parasitemia of 10^-2^ infected erythrocytes per ml of blood. Genomic DNA was extracted and nested PCR performed. Ten replicate amplifications were conducted per dilution to determine the lowest parasitemia level detected by nested PCR. All ten replicates were consistently positive with 10^4^ infected erythrocytes per ml of blood. In contrast, only four of 10 replicates were positive with 10^3^ infected erythrocytes per ml of blood. All replicates with 10^2^ infected erythrocytes per ml of blood were negative. The sensitivity of our nested PCR was determined to be 10^3^ infected erythrocytes per ml of blood which corresponded to a parasitemia as low as 0.000016%.

An immunoflurecence assay (IFA) was performed to detect *B*. *bovis* antibodies in deer sera. Briefly, cultures with 5% parasitized bovine erythrocytes were prepared by washing four times at 400xg in PBS. Infected erythrocyte pellets were suspended in PBS containing 1% bovine serum albumin (Fraction V) and thin smear microscope slides of infected erythrocytes prepared. The slides were air dried, covered, and stored at -80°C. Prior to performing the IFA, slides were warmed at room temperature in a desiccator jar for 30 min and fixed in cold acetone for 1 min. Sera were diluted 1:50 in PBS and applied to the antigen. The slides were incubated at 37°C in a humidity chamber for 30 min. After three successive washings in PBS, the slides were dried and exposed for 30 min with fluorescein conjugated Protein G (Rockland) diluted 1:5,000 in PBS to detect specific bovine and deer IgG. Protein G binds WTD immunoglobulin [24]. Slides were washed in PBS, dried and mounted in glycerol/PBS (1:1) and examined with an epifluorescence microscope.

Immunoblots were performed to identify *B*. *bovis* proteins recognize by specific antibodies in deer sera. Briefly, cultures with 30% parasitized bovine erythrocytes were washed with PBS and the pellets suspended in lysis buffer containing 50 mM Tris [pH 8], 5 mM EDTA, 1% Nonidet P-40, and Complete Protease Inhibitor (Roche) [20]. *Babesia bovis* or normal bovine erythrocyte antigens were resolved by SDS-PAGE, transferred to nitrocellulose membranes, and blocked with a solution containing 150 mM NaCl, 10 mM Tris-HCl, 1% polyvinylpyrrolidone, 0.2% Tween-20 and 5% non-fat milk (blocking solution). The membrane was cut into strips and incubated in sera from experimental WTD or control steers diluted 1:100 in blocking solution. The membrane strips were washed and incubated with a 1:20,000 dilution of Protein G conjugated with horseradish peroxidase in blocking solution. After washing, reactivity was detected by using HyGlo Quick Spray chemiluminescent substrate solution (Denville Scientific) and the membrane exposed to x-ray film.

Positive control steers were examined daily to determine when signs of bovine babesiosis occurred including alteration in body temperature, anorexia, anemia, weakness and depression. The infection of the steers was confirmed by 18S rRNA nested PCR amplification of DNA isolated from whole blood [15].

*Transfer of deer blood to susceptible recipient calves*: Whole blood (50 ml) from each WTD was collected in citrate phosphate dextrose anticoagulant and shipped on ice overnight to the U.S. Department of Agriculture-Agricultural Research Service-Animal Disease Research Unit (USDA-ARS-ADRU) facility in Pullman, WA. Deer blood was sub-inoculated into a susceptible splenectomized calf to determine if *B*. *bovis* had established an infection in WTD. To minimize the number of recipient animals, the blood from each WTD was inoculated separately into a single splenectomized recipient calf. Recipient calf #1 received blood from WTD challenged with *B*. *bovis* blood stabilate and calf #2 received blood from WTD challenged with the larval extract containing *B*. *bovis* sporozoites. Recipient animals were monitored daily for clinical signs of babesiosis for 35 days post-blood inoculation. Upon completion of this observation period, the susceptibility of the calves was tested by intravenous inoculation with *B*. *bovis*.

### Ethics Statement

This study was approved by the Institutional Animal Care and Use Protocol Committees of the University of Idaho, Moscow, Idaho and the Institutional Animal Care and Use Protocol Committees of the Knipling-Bushland United States Livestock Insects Research Laboratory, Kerrville, Texas, in accordance with institutional guidelines based on the U.S. National Institutes of Health (NIH) Guide for the Care and Use of Laboratory Animals. All splenectomy procedures were performed under sedation with xylazine and isoflurane inhalation, and all efforts were made to minimize suffering. All bovine and deer exposed with an exotic pathogen were sacrificed by injection intravenously of sodium pentobarbitone formulated for euthanasia, and the resulting sera and whole blood stored at −80°C.

## Results

In this study we tested the hypothesis that WTD are susceptible to infection and are competent reservoirs for *B*. *bovis*. Deer challenged with either *B*. *bovis* blood stabilate or larval supernatant extract containing sporozoites showed no evidence of transient or long-term infection, as determined by molecular and serological techniques. This was confirmed by the failure to cause infection upon transfer of blood from *B*. *bovis*-challenged WTD into susceptible, naïve calves.

### Challenge with *Babesia bovis* blood stabilate

**Fig 1:**
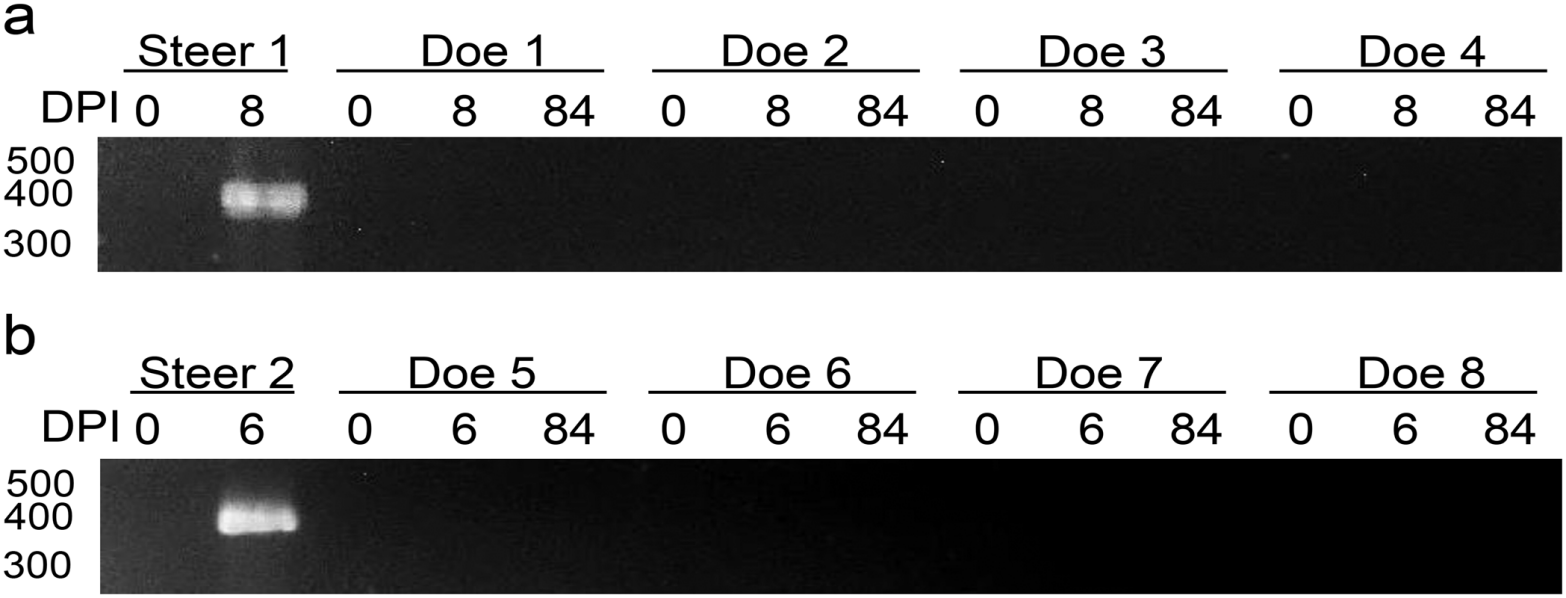
Evaluation of *Babesia bovis* infection by nested PCR. Amplicons were separated in 2% agarose by electrophoresis. White-tailed deer challenged with either *B*. *bovis* a) blood stabilate or b) a larval extract containing *B*. *bovis* sporozoites. Steer 1 and 2 control for the inocula. DPI: day post-inoculation. Molecular size is indicated on the left.

**Fig 2:**
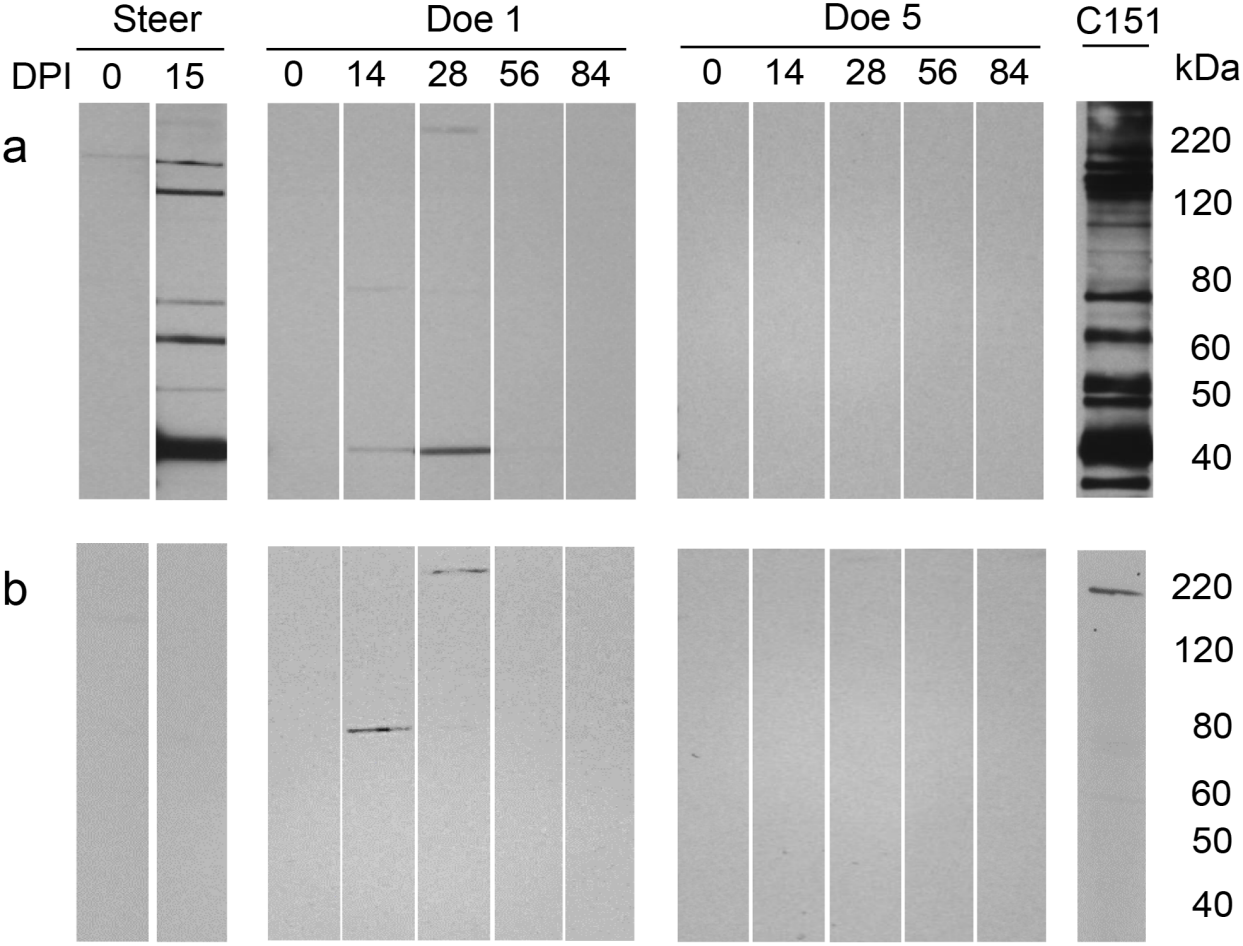
Detection of serum antibody reactivity to *B*. *bovis* infected erythrocyte and normal erythrocyte antigens after inoculation with a blood stabilate. Serum antibody from representative deer challenged with either *B*. *bovis* blood stabilate (Doe 1) or a larval extract containing *B*. *bovis* sporozoites (Doe 5) was evaluated by immunoblot. a) *B*. *bovis* infected erythrocyte antigen and b) normal bovine erythrocyte antigen. Steer: challenged with *B*. *bovis* blood stabilate and C151: bovine persistently infected with *B*. *bovis*. DPI: days post-inoculation.

Deer challenged with 10^9^ *B*. *bovis* infected erythrocytes showed no clinical signs of babesiosis throughout the experiment. Body temperature and PCV of the WTD remained within normal ranges of 38.4°C (±0.13) and 38.7% (± 2.1), respectively. Blood collected at multiple time points demonstrated that *B*. *bovis* failed to establish infection in deer, as determined by nested PCR (Fig 1a, Table 1). Immunoflurecence showed that three of four deer challenged with *B*. *bovis blood* stabilate developed antibodies against *B*. *bovis* and bovine erythrocytes. The same sera were tested by immunoblots and revealed that WTD developed antibody responses against proteins of 40, 70 and 220 kDa. Reactivity to the 40 kDa protein was observed only on immunoblots using *B*. *bovis* infected erythrocyte antigen (Fig 2a), but not on immunoblots using normal bovine erythrocyte antigen (Fig 2b). In contrast, reactivity to the 70 and 220 kDa proteins was also detected on immunoblots using normal bovine erythrocyte antigens (Fig 2b). Antibody responses against the inoculum were no longer detectable after 56 days post-inoculation.

**Table 1:** Evaluation of the white-tailed deer challenged with *Babesia bovis*.

	Week post-inoculation												
Group 1	0	1	2	3	4	5	6	7	8	9	10	11	12
Doe 1	N	n	n	n	n	n	n	n	n	n	n	n	n
Doe 2	n	n	n	n	n	n	n	n	n	n	n	n	n
Doe 3	n	n	n	n	n	n	n	n	n	n	n	n	n
Doe 4	n	n	n	n	n	n	n	n	n	n	n	n	n
Steer 1	n	N	P^SD^*										
	Week post-inoculation												
Group 2	0	1	2	3	4	5	6	7	8	9	10	11	12
Doe 5	n	n	n	n	n	n	n	n	n	n	n	n	n
Doe 6	n	n	n	n	n	n	n	n	n	n	n	n	n
Doe 7	n	n	n	n	n	n	n	n	n	n	n	n	n
Doe 8	n	n	n	n	n	n	n	n	n	n	n	n	n
Steer 2	n	P^SD^*											

White tailed deer challenged with *B*. *bovis*. Group 1: blood stabilate and group 2: a larval extract containing sporozoites. Nested PCR targeting 18S ribosomal RNA. N: negative; P: positive,

^SD^: Severe Clinical Disease and *: steers were euthanized.

Consistent with previous studies, the blood stabilate readily infected the control steer by 9 days post-inoculation as determined by PCR (Fig 1a) and severe clinical signs of bovine babesiosis including elevation of body temperature (40.7°C) and a decline in the PCV to 29%. The steer was euthanized to minimize animal suffering. Immunoflurecence demonstrated that the steer had an antibody response to *B*. *bovis* antigens. Immunoblots demonstrated that the steer responded to multiple *B*. *bovis* antigens ranging from 40 kDa to 120 kDa (Fig 2a and 2b).

### Challenge with larval extract supernatant containing *Babesia bovis* sporozoites

Infectivity of a larval extract containing 10^5^ *B*. *bovis* sporozoites was confirmed by intravenous inoculation of a control steer with the same inoculum used for the WTD challenge. The steer was PCR positive at 6 days post-sporozoite inoculation (Fig 1b). The larval extract containing *B*. *bovis* sporozoites caused rapid severe clinical signs of bovine babesiosis in the steer including elevation of body temperature (40.8°C) and a decline in the PCV to 23%. To minimize animal suffering, the steer was euthanized.

WTD challenged with larval extract containing 10^5^ *B*. *bovis* sporozoites showed no evidence of infection. Body temperature as well as PCV of the deer remained within normal ranges of 38.9°C (± 0.09) and 38.3% (±3.5), respectively. PCR amplification of blood collected weekly from the deer indicated that *B*. *bovis* sporozoites within the larval extract failed to establish infection (Table 1). Throughout the experiment, none of the deer in this group showed evidence of an antibody response against *B*. *bovis* by either IFA or immunoblots using *B*. *bovis* infected erythrocyte antigen (Fig 2a) or normal bovine erythrocyte antigen (Fig 2b).

### Blood transfer from deer into susceptible splenectomized cattle

**Fig 3:**
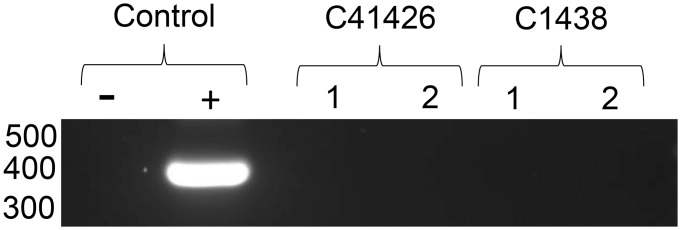
Analysis of naïve susceptible bovines that received blood transferred from deer challenged with *B*. *bovis* by nested PCR. Calves 41426 and 1438 received blood from deer challenged with either *B*. *bovis* blood stabilate or a larval extract supernatant containing *B*. *bovis* sporozoites, respectively. Amplicons were separated in 2% agarose by electrophoresis for visualization. Genomic DNA from 1: pre-inoculation and 2: five weeks post-inoculation. Molecular size is indicated on the left.

Blood from WTD challenged with either *B*. *bovis* infected erythrocytes or a larval extract containing sporozoites failed to infect susceptible cattle. Clinical signs of babesiosis were not observed in the splenectomized calves throughout the experiment (data not shown). PCR results during the five weeks post-inoculation showed no evidence of infection (Fig 3). To test the susceptibility of the calves, *B*. *bovis* stabilate was inoculated intravenously and severe clinical signs of bovine babesiosis were observed at day 10 post-inoculation including body temperature exceeding 40.6°C. During acute infection, parasites were detected by Giemsa blood smear or PCR amplification and calves were euthanized to minimized animal suffering.

## Discussion

The wildlife-livestock interface is a restriction to the success of controlling and eradicating diseases of domestic animals [25–29] highlighting the necessity to understand the importance of wildlife in the epidemiology and maintenance of infectious diseases of livestock. This is illustrated by the incidence of tick-transmitted bovine theileriosis in Africa. The disease is caused by *Theileria parva* and is typically transmitted from infected to susceptible cattle via ticks. However, Cape buffalo (*Syncerus caffer*) are known competent reservoirs for *T*. *parva* and occupies overlapping habitats with cattle, providing a continuous source of parasite for tick transmission [25,28,30]. Biological vectors that acquire infection while feeding on buffalo can transmit the pathogen to either buffalo or cattle causing significant economic loss to the livestock industry.

It is critical to understand if wildlife can be a source of pathogens for transmission to livestock. The lack of knowledge regarding pathogens that infect wildlife and the difficulties with disease surveillance of wildlife create obstacles to detection and monitoring for organisms that can cause significant disease in livestock. Recent reports suggested that free-ranging WTD commingling with livestock could be a source of tick-borne pathogens that cause bovine babesiosis [15–17]. However, it remained unclear whether WTD were indeed competent reservoirs. In this study, we tested the hypothesis that *B*. *bovis* blood stabilate or a larval extract supernatant containing sporozoites were infective to WTD. The collective data does not support an epidemiological role for WTD in the maintenance of *B*. *bovis*. This conclusion is supported by the failure of *B*. *bovis* to establish an infection in WTD by either *B*. *bovis* blood or tick stages. Nested PCR results were consistently negative at multiple time points post-inoculation, suggesting there was no transient infection. Blood from WTD challenged with *B*. *bovis* failed to infect naïve susceptible recipient calves, confirming that WTD are not a reservoir for *B*. *bovis*.

A previous attempt also failed to infect WTD with bovine babesial parasites upon inoculation of spleen-intact or splenectomized deer with infected blood from a naturally infected bovine [14]. However, the level of parasitemia used in the challenge study was undetectable by light microscopy of stained thin smears. With our challenge strain, a minimum dose sufficient to infect cattle and cause severe disease is ten *B*. *bovis* infected erythrocytes [21]. In order to definitively determine whether or not WTD are susceptible to *B*. *bovis* infection, we challenged the animals with >10^8^ more parasites needed to sufficiently infect cattle. Although we challenged WTD with a high dose, the deer showed no evidence of infection suggesting that WTD are not susceptible to *B*. *bovis* infection. Our results shed light on the inability of blood and serum from WTD to support *in vitro* culture of *B*. *bovis* [31].

Attempts to transmit babesial parasites using infected ticks harvested from a natural infected animal failed to infect deer [14]. Persistently infected calves harbor dramatically lower levels of parasites in peripheral blood, resulting in low larval infection rates from eggs of *R*. *microplus* females allowed to acquisition feed [3]. The infected larvae used by Kuttler et al (1972) to infest WTD were obtained from female *R*. *annulatus* fed on a clinically negative animal naturally infected with babesial parasites. Further, larval feeding rates of cattle fever ticks on WTD can be variable depending on the level of resistance to tick infestation and grooming [13,32]. To overcome these issues, we produced a larval extract supernatant containing *B*. *bovis* sporozoite from infected *R*. *microplus* larvae and inoculated them into WTD. This approach allowed us to quantify the amount of *B*. *bovis* and ensure a high dose was delivered to each animal. The results provide additional evidence that WTD are not susceptible to *B*. *bovis* infection.

A salient distinction between the current study and previous *Babesia* spp. surveillance studies is our use of tick-naïve WTD. Deer challenged with a single exposure responded to a single *B*. *bovis* protein, but because there was not continuous stimulation by an established infection, antibodies against *B*. *bovis* were no longer detected after 6 weeks. In contrast, acute infection in cattle resulted in a response to multiple *B*. *bovis* proteins. A single inoculum of 10^5^ *B*. *bovis* parasites contained within a larval extract supernatant failed to stimulate a deer immune response demonstrating that *B*. *bovis* did not establish infection in deer. In contrast, free-ranging WTD captured at a single time point likely had multiple exposures to various tick species, each providing opportunities for exposure to the parasite delivered via tick saliva during feeding. Such parasite exposure would elicit an immune response in WTD, explaining the detection of anti-*B*. *bovis* and *B*. *bigemina* antibodies in these samples. Tick salivary proteins can modulate the mammalian immune response to the feeding tick, enabling larvae to feed for days [33]. Thus, the parasite can be continuously inoculated into the blood stream via saliva. Detection of DNA isolated from peripheral blood may be possible without an infection necessarily being established due to the accumulation of parasites in the blood. Indeed, Cantu et al (2009) observed that the frequency of *Babesia* spp. detection by serology or PCR was elevated in scenarios where exposure to cattle ticks was increased [17]. Pharmacologically bioactive tick proteins may enhance *Babesia* transmission. However, the results from the current study using a larval extract containing *B*. *bovis* sporozoites and a previous study performed by Kuttler et al (1972) that attempted to transmit parasites via on-animal infestation with infected *Rhipicephalus* larvae demonstrated no evidence of *Babesia* infection in WTD. Together, these collective results indicate that these free-ranging WTD do not present a risk for *B*. *bovis* transmission.

In conclusion, we demonstrated that WTD are not transiently infected with and are not a reservoir of *B*. *bovis*. These findings provide evidence that WTD are not an epidemiological threat for maintenance of bovine babesiosis to livestock. However, it is possible that co-evolution between the mammalian host-vector-pathogen may influence parasite adaptation to wildlife and these animals could become potential reservoirs of parasites. Therefore, it is important for the United States to maintain continuous surveillance of wildlife, including WTD, to provide control measurement for preventing bovine babesiosis emerging in areas free of infectious disease.

## References

[1] De Moraes CM, Stanczyk NM, Betz HS, Pulido H, Sim DG, Read AF, et al Malaria-induced changes in host odors enhance mosquito attraction. Proc Natl Acad Sci U S A. 2014; 111: 11079–11084. 1405617111 [pii]; 10.1073/pnas.1405617111 24982164 PMC4121820

[2] Ueti MW, Tan Y, Broschat SL, Castaneda Ortiz EJ, Camacho-Nuez M, Mosqueda JJ, et al Expansion of variant diversity associated with a high prevalence of pathogen strain superinfection under conditions of natural transmission. Infect Immun. 2012; 80: 2354–2360. IAI.00341-12 [pii]; 10.1128/IAI.00341-12 22585962 PMC3416468

[3] Howell JM, Ueti MW, Palmer GH, Scoles GA, Knowles DP. Persistently infected calves as reservoirs for acquisition and transovarial transmission of *Babesia bovis* by *Rhipicephalus* (*Boophilus*) *microplus* . J Clin Microbiol. 2007; 45: 3155–3159. JCM.00766-07 [pii]; 10.1128/JCM.00766-07 17687016 PMC2045367

[4] Howell JM, Ueti MW, Palmer GH, Scoles GA, Knowles DP. Transovarial transmission efficiency of *Babesia bovis* tick stages acquired by *Rhipicephalus* (*Boophilus*) *microplus* during acute infection. J Clin Microbiol. 2007; 45: 426–431. JCM.01757-06 [pii]; 10.1128/JCM.01757-06 17166964 PMC1829031

[5] Futse JE, Brayton KA, Dark MJ, Knowles DP Jr., Palmer GH. Superinfection as a driver of genomic diversification in antigenically variant pathogens. Proc Natl Acad Sci U S A. 2008; 105: 2123–2127. 0710333105 [pii]; 10.1073/pnas.0710333105 18252822 PMC2538888

[6] Suarez CE, Noh S. Emerging perspectives in the research of bovine babesiosis and anaplasmosis. Vet Parasitol. 2011; 180: 109–125. S0304-4017(11)00385-2 [pii]; 10.1016/j.vetpar.2011.05.032 21684084

[7] Bock R, Jackson L, de VA, Jorgensen W. Babesiosis of cattle. Parasitol. 2004; 129 Suppl: S247–S269. 15938514 10.1017/s0031182004005190

[8] Lopez M, Figueroa JV, Ramos JA, Mosqueda JJ, Rojas E, Vega CA, et al Infection and seroconversion of susceptible animals introduced into a babesiosis endemic area. Ann N Y Acad Sci. 2008; 1149: 131–135. NYAS1149072 [pii]; 10.1196/annals.1428.072 19120191

[9] Phillips PL, Welch JB, Kramer M. Development of a spatially targeted field sampling technique for the southern cattle tick, *Rhipicephalus microplus*, by mapping white tailed deer, *Odocoileus virginianus*, habitat in South Texas. J Insect Sci. 2014; 14: 88 14.1.88 [pii]; 10.1093/jis/14.1.88 25368044 PMC4213131

[10] Busch JD, Stone NE, Nottingham R, Araya-Anchetta A, Lewis J, Hochhalter C, et al Widespread movement of invasive cattle fever ticks (*Rhipicephalus microplus*) in southern Texas leads to shared local infestations on cattle and deer. Parasit Vectors. 2014; 7: 188 1756-3305-7-188 [pii]; 10.1186/1756-3305-7-188 24742041 PMC4022356

[11] Lohmeyer KH, Pound JM, May MA, Kammlah DM, Davey RB. Distribution of *Rhipicephalus* (*Boophilus*) *microplus* and *Rhipicephalus* (*Boophilus*) *annulatus* (Acari: Ixodidae) infestations detected in the United States along the Texas/Mexico border. J Med Entomol. 2011; 48: 770–774. 21845935 10.1603/me10209

[12] Pound JM, George JE, Kammlah DM, Lohmeyer KH, Davey RB. Evidence for role of white-tailed deer (Artiodactyla: Cervidae) in epizootiology of cattle ticks and southern cattle ticks (Acari: Ixodidae) in reinfestations along the Texas/Mexico border in south Texas: a review and update. J Econ Entomol. 2010; 103: 211–218. 20429430 10.1603/EC09359

[13] Cooksey LM, Davey RB, Ahrens EH, George JE. Suitability of white-tailed deer as hosts for cattle fever ticks (Acari: Ixodidae). J Med Entomol. 1989; 26: 155–158. 2724312 10.1093/jmedent/26.3.155

[14] Kuttler KL, Graham OH, Johnson SR, Trevino JL. Unsuccessful attempts to establish cattle *Babesia* infections in white-tailed deer. J Wildl Dis. 1972; 8: 63–66. 4621443 10.7589/0090-3558-8.1.63

[15] Holman PJ, Carroll JE, Pugh R, Davis DS. Molecular detection of *Babesia bovis* and *Babesia bigemina* in white-tailed deer (*Odocoileus virginianus*) from Tom Green County in central Texas. Vet Parasitol. 2011; 177: 298–304. S0304-4017(10)00702-8 [pii]; 10.1016/j.vetpar.2010.11.052 21194841

[16] Ramos CM, Cooper SM, Holman PJ. Molecular and serologic evidence for *Babesia bovis*-like parasites in white-tailed deer (*Odocoileus virginianus*) in south Texas. Vet Parasitol. 2010; 172: 214–220. S0304-4017(10)00283-9 [pii]; 10.1016/j.vetpar.2010.05.004 20605333

[17] Cantu C, Ortega-S JA, Garcia-Vazquez Z, Mosqueda J, Henke SE, George JE. Epizootiology of *Babesia bovis* and *Babesia bigemina* in free-ranging white-tailed deer in northeastern Mexico. J Parasitol. 2009; 95: 536–542. GE-1648 [pii]; 10.1645/GE-1648.1 19642800

[18] Cantu A, Ortega-S JA, Mosqueda J, Garcia-Vazquez Z, Henke SE, George JE. Immunologic and molecular identification of *Babesia bovis* and *Babesia bigemina* in free-ranging white-tailed deer in northern Mexico. J Wildl Dis. 2007; 43: 504–507. 43/3/504 [pii]; 10.7589/0090-3558-43.3.504 17699089

[19] Brayton KA, Lau AO, Herndon DR, Hannick L, Kappmeyer LS, Berens SJ, et al Genome sequence of *Babesia bovis* and comparative analysis of apicomplexan hemoprotozoa. PLoS Pathog. 2007; 3: 1401–1413. 07-PLPA-RA-0191 [pii]; 10.1371/journal.ppat.0030148 17953480 PMC2034396

[20] Awinda PO, Mealey RH, Williams LB, Conrad PA, Packham AE, Reif KE, et al Serum antibodies from a subset of horses positive for *Babesia caballi* by competitive enzyme-linked immunosorbent assay demonstrate a protein recognition pattern that is not consistent with infection. Clin Vaccine Immunol. 2013; 20: 1752–1757. CVI.00479-13 [pii]; 10.1128/CVI.00479-13 24049108 PMC3837787

[21] Goff WL, Johnson WC, Cluff CW. *Babesia bovis* immunity. In vitro and in vivo evidence for IL-10 regulation of IFN-gamma and iNOS. Ann N Y Acad Sci. 1998; 849: 161–180. 9668462 10.1111/j.1749-6632.1998.tb11046.x

[22] Sondgeroth KS, McElwain TF, Ueti MW, Scoles GA, Reif KE, Lau AO. Tick passage results in enhanced attenuation of *Babesia bovis* . Infect Immun. 2014; 82: 4426–4434. IAI.02126-14 [pii]; 10.1128/IAI.02126-14 25114111 PMC4187863

[23] Oosthuizen MC, Zweygarth E, Collins NE, Troskie M, Penzhorn BL. Identification of a novel *Babesia* sp. from a sable antelope (Hippotragus niger Harris, 1838). J Clin Microbiol. 2008; 46: 2247–2251. JCM.00167-08 pii]; 10.1128/JCM.00167-08 18508943 PMC2446884

[24] Kramsky JA, Manning EJ, Collins MT. Protein G binding to enriched serum immunoglobulin from nondomestic hoofstock species. J Vet Diagn Invest. 2003; 15: 253–261. 12735347 10.1177/104063870301500306

[25] Walker JG, Klein EY, Levin SA. Disease at the wildlife-livestock interface: acaricide use on domestic cattle does not prevent transmission of a tick-borne pathogen with multiple hosts. Vet Parasitol. 2014; 199: 206–214. S0304-4017(13)00599-2 [pii]; 10.1016/j.vetpar.2013.11.008 24315187

[26] Caron A, Miguel E, Gomo C, Makaya P, Pfukenyi DM, Foggin C, et al Relationship between burden of infection in ungulate populations and wildlife/livestock interfaces. Epidemiol Infect. 2013;141: 1522–1535. S0950268813000204 [pii]; 10.1017/S0950268813000204 23442901 PMC9151594

[27] Berggoetz M, Schmid M, Ston D, Wyss V, Chevillon C, Pretorius AM, et al Tick-borne pathogens in the blood of wild and domestic ungulates in South Africa: interplay of game and livestock. Ticks Tick Borne Dis. 2014; 5: 166–175. S1877-959X(13)00121-0 [pii]; 10.1016/j.ttbdis.2013.10.007 24418761

[28] Berggoetz M, Schmid M, Ston D, Wyss V, Chevillon C, Pretorius AM, et al Protozoan and bacterial pathogens in tick salivary glands in wild and domestic animal environments in South Africa. Ticks Tick Borne Dis. 2014; 5: 176–185. S1877-959X(13)00116-7 [pii]; 10.1016/j.ttbdis.2013.10.003 24378080

[29] Githaka N, Konnai S, Bishop R, Odongo D, Lekolool I, Kariuki E, et al Identification and sequence characterization of novel *Theileria* genotypes from the waterbuck (*Kobus defassa*) in a *Theileria parva*-endemic area in Kenya. Vet Parasitol. 2014; 202: 180–193. S0304-4017(14)00147-2 [pii]; 10.1016/j.vetpar.2014.02.056 24690249

[30] Odongo DO, Ueti MW, Mwaura SN, Knowles DP, Bishop RP, Scoles GA. Quantification of *Theileria parva* in *Rhipicephalus appendiculatus* (Acari: Ixodidae) confirms differences in infection between selected tick strains. J Med Entomol. 2009; 46: 888–894. 19645294 10.1603/033.046.0422

[31] Holman PJ, Waldrup KA, Droleskey RE, Corrier DE, Wagner GG. *In vitro* growth of *Babesia bovis* in white-tailed deer (*Odocoileus virginianus*) erythrocytes. J Parasitol. 1993; 79: 233–237. 8459334

[32] Davey RB. Failure of white-tailed deer, *Odocoileus virginianus* L., to sustain a population of cattle ticks, *Boophilus annulatus* (Say), through successive generations. J Parasitol. 1990; 76: 356–359. 2352065

[33] Wikel S. Ticks and tick-borne pathogens at the cutaneous interface: host defenses, tick countermeasures, and a suitable environment for pathogen establishment. Front Microbiol. 2013; 4: 337 10.3389/fmicb.2013.00337 24312085 PMC3833115

